# COVID-19 affects healthy pediatricians more than pediatric patients

**DOI:** 10.1017/ice.2020.139

**Published:** 2020-04-16

**Authors:** Nima Rezaei

**Affiliations:** Research Center for Immunodeficiencies, Children’s Medical Center, Tehran University of Medical Sciences, Tehran, Iran; Department of Immunology, School of Medicine, Tehran University of Medical Sciences, Tehran, Iran; Network of Immunity in Infection, Malignancy and Autoimmunity (NIIMA), Universal Scientific Education and Research Network (USERN), Tehran, Iran

*To the Editor*—Coronavirus disease 2019 (COVID-19) emerged in China in late December and has spread rapidly throughout the world. The World Health Organization (WHO) considers it a pandemic. In ~4 months from start of this outbreak, as of April 4, 2020, >1,000,000 patients had been affected.^1^

Meanwhile, children appear less likely to be affected than adults; among the affected children, most have mild symptoms and some are even asymptomatic.^2-4^ The Chinese Center for Disease Control and Prevention has reported that among ~70,000 cases, <1% were aged <10 years.^5^

The first official COVID-19 case in Iran was announced on February 19, 2020. On March 16, 2020, 4 weeks later, the daily situation report on COVID-19 showed that <1% of ~5,000 confirmed cases were children aged <10 years.^6^ The Children’s Medical Center in Iran has 348 beds for pediatric patients, with bed occupancy rate of 94.7% and an average length of stay of 3.3 days. During the 4-week period after the first identified case in the country, only 3 positive COVID-19 cases were identified, for an estimated frequency of 1 per 1,000 admitted pediatric patients. Meanwhile, among 60 pediatricians working in this hospital, 5 were positive for COVID-19 by real-time reverse transcription polymerase chain reaction (rRT-PCR), for a prevalence of 8.3%, which was much higher than the frequency of COVID-19 in general population (~2 per 10,000) during this 4-week period.

One hypothesis is that aging is a condition associated with inflammation, whereas children might have an immature anti‐inflammatory response. Possibly, therefore, an increased inflammatory reaction is expected in adult subjects compared to children.^7^

Not only the pediatricians but also other healthcare professionals are at a higher risk of infection with COVID-19.^8^ A report from Italy revealed that ~20% of healthcare professionals had become infected.^9^ Healthcare professionals are at the frontline of fighting COVID-19 in hospitals, where they are in contact with visiting patients and their parents who are potentially carriers of SARS-CoV-2, even if they are not infected. Indeed these healthcare professionals are under excessive workload pressure and psychological distress during the pandemic, which can lead to caregiver burnout.^10^ Pediatricians, especially those who have children at home, not only have concerns about passing the infection to their children but also about not caring enough for their children during quarantine period, considering school closures and social distancing policies.^8^ Therefore, healthcare systems should be very careful to address the physical and mental health of healthcare professionals. Easy access to personal protective equipment, especially for those who are visiting patients with COVID-19, and psychological support for those who are losing their patients and colleagues, especially for those who cannot see their family members for long periods, are necessary.

## References

[1] Johns Hopkins Coronavirus Resource Center website. https://coronavirus.jhu.edu/map.html. Accessed April 4, 2020.

[2] Dong Y , Mo X , Hu Y , et al. Epidemiology of COVID-19 among children in China. Pediatrics 2020 March 16. doi: 10.1542/peds.2020-0702. 32179660

[3] Cruz A , Zeichner S . COVID-19 in children: initial characterization of the pediatric disease. Pediatrics 2020 March 16. doi: 10.1542/peds.2020-0834. 32179659

[4] Lu X , Zhang L , Du H , et al. SARS-CoV-2 infection in children. N Engl J Med 2020 Mar 18. doi: 10.1056/NEJMc2005073. PMC7121177 32187458

[5] Wu Z , McGoogan JM . Characteristics of and important lessons from the coronavirus disease 2019 (COVID-19) outbreak in China: summary of a report of 72 314 cases from the Chinese Center for Disease Control and Prevention. JAMA 2020 Feb 24. doi: 10.1001/jama.2020.2648. 32091533

[6] COVID-19 daily epidemiology journal. Iranian Ministry of Health and Medical Education website. http://corona.behdasht.gov.ir/files/site1/files/Factsheet_4._26.12_-_En.pdf. Published March 16, 2020. Accessed April 15, 2020.

[7] Saghazadeh A , Rezaei N . Immune-epidemiological parameters of the novel coronavirus—a perspective. Expert Rev Clin Immunol 2020 Apr 1. doi: 10.1080/1744666X.2020.1750954. PMC7157951 32237901

[8] Lo D . COVID-19: protecting healthcare workers. Lancet 2020;395(10228):922. doi: 10.1016/S0140-6736(20)30644-9. PMC7138074 32199474

[9] Remuzzi A , Remuzzi G . COVID-19 and Italy: what next? Lancet 2020 Mar 13. doi: 10.1016/S0140-6736(20)30627-9. PMC7102589 32178769

[10] Moazzami B , Razavi-Khorasani N , Dooghaie Moghadam A , Farokhi E , Rezaei N . COVID-19 and telemedicine: immediate action required for maintaining healthcare providers well-being. J Clin Virol 2020 Apr 4. doi: 10.1016/j.jcv.2020.104345. PMC7129277 32278298

